# Innovative models for enhanced student adaptability and performance in educational environments

**DOI:** 10.1371/journal.pone.0307221

**Published:** 2024-09-06

**Authors:** Lanbo Liu, Lihong Wan

**Affiliations:** 1 Tianjin University of Technology and Education, Tianjin, China; 2 School of Marxism, Guangdong Polytechnic Normal University, Guangzhou, Guangdong, China; BRAC Business School, BRAC University, BANGLADESH

## Abstract

In the domain of adaptable educational environments, our study is dedicated to achieving three key objectives: forecasting the adaptability of student learning, predicting and evaluating student performance, and employing aspect-based sentiment analysis for nuanced insights into student feedback. Using a systematic approach, we commence with an extensive data preparation phase to ensure data quality, followed by applying efficient data balancing techniques to mitigate biases. By emphasizing higher education or educational data mining, feature extraction methods are used to uncover significant patterns in the data. The basis of our classification method is the robust WideResNeXT architecture, which has been further improved for maximum efficiency by hyperparameter tweaking using the simple Modified Jaya Optimization Method. The recommended WResNeXt-MJ model has emerged as a formidable contender, demonstrating exceptional performance measurements. The model has an average accuracy of 98%, a low log loss of 0.05%, and an extraordinary precision score of 98.4% across all datasets, demonstrating its efficacy in enhancing predictive capacity and accuracy in flexible learning environments. This work presents a comprehensive helpful approach and a contemporary model suitable for flexible learning environments. WResNeXt-MJ’s exceptional performance values underscore its capacity to enhance pupil achievement in global higher education significantly.

## Introduction

“Education 2.0” is a term used to describe the great use of digital technology in early twenty-first century institutions. Then Education 3.0 arrived, as a more user-generated internet became more accessible. Students have fast access to information, may study from anywhere, and effective lines of interaction with teachers and peers. This shift marked notable advancement. Students’ fast access to many information sources beyond the conventional teacher-student dynamic results from the networked education paradigm [1]. As the fourth wave of industrialization rolls in, teachers are pushing “Education 4.0,” a revolutionary approach to education that improves learning opportunities using new technologies.

Data mining’s possible educational uses have attracted fresh attention in recent years. Data mining is systematically exploring massive datasets for functional patterns or information. Multiple methods exist for categorizing patterns and relationships that are constantly changing, as stated by DM [2]. Educational data mining (EDM) addresses educational issues by analyzing student information, course records, exam results, student participation, and inquiry frequency. EDM could shed light on trends, academic achievement, flexibility, and the learning environment. One such is the relationship between greater student retention rates [3] and EDM’s usage for early detection of at-risk pupils.

Machine Learning (ML) applications have been advancing fast in marketing, finance, and health [4]. A subset of artificial intelligence, ML allows machines to make predictions on their own. The educational field is undergoing tectonic shifts in teaching and learning methodologies due to the adoption of ML-based approaches. This particular case displays a high degree of the extent to which ML could radically transform education. It identifies complex associations. By means of their perceptive analysis, ML algorithms enable the organization of enormous volumes of student data and create a methodological panopticon depending on the features of every student [5].

In traditional schools, courses are mostly not self-paced; therefore, there must be some flexibility, as seen in [6] because students have diverse learning styles. Also, meet this need for adaptability by learning at the level of every student-learning activity velocity [7]. Machine learning algorithms enable better subject insight, and students get an interactive way of education here by tracking student progress to find weak areas in the syllabus that need changing [8].

One of the forms associated with AI, sentiment analysis extracts information from textual content to comprehend what users are feeling and thinking [8]. According to [9], sentiment analysis offers valuable insights into students’ mental health, classroom engagement, and openness to course content. By quickly addressing student concerns, sentiment analysis of student writing, online comments, and debates may assist instructors in establishing a welcoming classroom environment. Another application of machine learning, predictive analytics, has the potential to improve academic performance by predicting student outcomes and identifying individuals who are at risk of failing [10].

Improved course design and resource management aid schools; early intervention by instructors helps pupils. Machine learning transforms the dynamics of teaching and learning by improving general educational processes and meeting the needs of every learner [11]. Though it is still in its infancy, the educational technology revolution has the potential to create a more fair and efficient system that meets every student’s particular need. By delving into groundbreaking findings, researchers, academics, and decision-makers may unlock machine learning’s full potential, bringing about a new age that values academic success, mental wellness, and uniqueness. AI and ML have recently attracted much attention in online education research because they provide appropriate methods for regularly processing online education’s massive volumes of data [12].

### Motivation and contributions

Driven by the conviction that combining machine learning with education will significantly influence future learning practices, our investigation seeks to explore the potential benefits of this integration. The educational landscape is dynamic and constantly evolving to meet student’s diverse needs and goals. Motivated by a strong desire to address crucial facets of education, we emphasize the importance of adaptation for inclusive and prosperous teaching, given the variety of student backgrounds and learning preferences [13]. Additionally, recognizing the significance of the rapidly evolving technological world, we focus on ensuring that technology enhances the learning experience [14]. Lastly, as sentiment analysis sheds light on how emotions impact learning [15], it becomes a valuable tool for comprehending academic achievement and satisfaction.

Our overarching goal is to provide an educational platform that merges the advanced capabilities of DL algorithms with revolutionized teaching and learning approaches from the past. The methodology integrates aspect-based sentiment analysis, adaptability, and academic accomplishment, all substantiated by large datasets. We aim to show individuals prompt answers to their questions about education and provide a group forum for exchanging ideas. Envisioning a future where education is more efficient, inclusive, and personalized to each student’s requirements, we anticipate achieving this through integrating DL approaches.

The ultimate goal is to provide students with a flexible, engaging learning environment that transforms their learning approach, facilitating academic and personal success. By utilizing the power of education and technology, we can contribute to creating a better future for students worldwide.

**Contributions:** Our research provides an extensive array of scientific breakthroughs, principally centered on extending the potential of EDM and DL inside the educational sphere, creating an innovative approach to improving instruction.

- Understanding the factors influencing student performance (family aspects, student learning behaviors, and teaching methodologies) is a fundamental aspect of this study. The primary focus is establishing effective connections that analyze student performance, adaptability, and feedback analysis.
- This study pioneers creating a hybrid lightweight model, namely WideResNeXt, combined with GRU (WResNeXt-G), capable of handling sequential and temporal aspects of incoming diverse big data. Additionally, it explores patterns in learning behaviors and assesses the adaptability of students based on evolving conditions, even with limited data.
- To address biases, regularities, and variations in a large volume of data, the hyperparameters of the hybrid method WResNeXt-G are fine-tuned using the Modified Jaya Optimization Algorithm. The resulting model is referred to as WResNeXt-GMJ. When the number of dimensions or characteristics in the input data changes or rises, the Jaya Algorithm tunes the values of the parameters.
- To ensure the model is efficient, it is compared to models in the literature and evaluated using metrics including log loss, evaluation metrics, confusion matrix validation, and statistical analysis to verify hypotheses. Our model demonstrates superior performance across all validation and evaluation metrics.
- The Model achieves high accuracy and precision, ranging from 95.9% to 98.7%, even with variations in data values. It also exhibits optimal execution time/computational time compared to other methods across different dataset scenarios.

## Related work

As technology advances open the way for the development of online education systems, current activities emphasize their critical necessity. These systems support distant or online education, which aligns with the current digitalization trend. Students must overcome the problem of adjusting to online learning. As part of our investigation, we thoroughly examined previous academic studies in this field. The critical realization that learning flexibility significantly influences students’ feedback and performance scores highlights the requirement for a responsive and adaptable educational strategy. The summarized view of related work is shown in Table 1, and subsequent sections display detailed literature.

**Table 1:** Summary of studies and limitations.

Ref	Problem	Method Used	Achievement	Limitations
[16]	Adaptability	Random Forest	89.63% accuracy	Limited generalizability due to dataset specificity.
[17]	Flexibility	RF, IBK	Accurate forecasting of mobile learning apps uptake	Influence of diverse user demographies and environmental factors.
[18]	Adaptability	SVM, DT, DNN	Creation of a feature model for predicting pass rates	Potential oversight of relevant student traits in the feature model.
[19]	Adaptability	Ensemble Learning Techniques	Explored solutions for addressing student dropout rates	Incomplete coverage of multifaceted factors contributing to student dropout.
[20]	Adaptability	Scalable XGBoost	Enhanced consistency in student performance	Scalability and generalizability requiring further validation across diverse educational contexts.
[21]	Sentiment Analysis	Algorithmic Supervision, SVM, NB, ID3	Evaluated instructor effectiveness based on sentiment analysis	Subjectivity in interpreting sentiment outcomes from student feedback.
[22]	Sentiment Analysis	Teaching Senti-Lexicon, SVM, NB, ID3	Showcased advantages of Teaching Senti-Lexicon	Efficacy of Teaching Senti-Lexicon dependent on lexicon specificity and contextual relevance.
[23]	Sentiment Analysis	Decision Trees	Early prediction of academic failure using student self-evaluations	Predictive accuracy influenced by complexity in student behaviors.
[24]	Sentiment Analysis	Decision Trees	Generated yearly assessment reports based on student comments	Reliance on comments and performance metrics may overlook nuanced teacher performance indicators.
[25]	Sentiment Analysis	Lexicon-Based Technique	Provided visual insights into instructors’ performance	Potential oversimplification of sentiment analysis in educational feedback.
[26]	Sentiment Analysis	Faculty Evaluation System	Demonstrated the utility of sentiment models	Applicability of sentiment models may vary based on feedback structures and contextual nuances.
[27]	Sentiment Analysis	Three Methods	Identified categories in SMS texts	accuracy of category identification influenced by linguistic diversity and brevity in SMS texts.
[28]	Sentiment Analysis	Naive Bayes	Classified comments in English and Filipino languages	Effectiveness of sentiment classification may vary across different languages.
[29]	Sentiment Analysis	SVM	Evaluated sentiment on an educational dataset	model accuracy dependent on the representativeness of the educational dataset.
[30]	Sentiment Analysis	Aspect-Based Sentiment Evaluation	Identified and categorized features based on sentiment	Efficacy of aspect-based sentiment evaluation tied to the granularity of feature identification.
[31]	Sentiment Analysis	Dependency Connections, SVM	Achieved noteworthy F-scores in aspect extraction and emotion detection	Context-dependent F-scores influenced by complexity in student sentiments.
[32]	Performance	Association Rule Mining	Extracted useful information from large datasets	Computational intensity and time-consuming processes associated with Association Rule Mining.
[33]	Performance	Various Classifiers	Predicted student performance	Accuracy of predictions influenced by classifier choice and hyperparameters.
[34]	Performance	Decision Trees (BfTree, J48, CART)	Predicted student achievement	Differences in Accuracy among decision tree algorithms affecting prediction reliability.

### Adaptability concept in education

The author in [16] collects data from students at various academic levels using both physical and online surveys. Sociodemographic information is gathered from the study, and artificial intelligence techniques are applied to assess how well-suited the students are for online learning. The most accurate approach is the Random Forest (RF) classifier, which has an accuracy rating of 89.63Moreover, [17] proposes a hybrid strategy to identify crucial parameters impacting the acceptability of mobile learning apps by combining TAM with additional TUT model components. Anticipated relationships between model components are explored by applying machine learning techniques. Among all the algorithms, IBK and RF have shown the highest level of performance in forecasting the adoption of learning via mobile devices.

The author of [18] investigates particular student traits to forecast online learning pass rates. The study’s two main goals are predicting student pass rates and figuring out the best machine-learning method to find essential student characteristics that affect learning. Three algorithms are used to create a feature model: SVM, DT, and DNN. Meanwhile, the researcher explores the critical topic of rates of dropping out of online courses and education [19]. The research aims to predict viable resolutions to the problem of student attrition.

Ensemble learning techniques are used in different research [20] to produce creative solutions in various fields. The authors provide a novel PFA method that enhances student performance’s reliability by drawing from multiple models, including Boost, RF, and XGBoost. According to extensive testing on three separate datasets, scalable XGBoost outperforms other models and performs far better than the original PFA method in performance prediction.

### Aspect-based sentiment evaluation in education

In the academic literature, sentiment analysis has been the focus of many research investigations, categorizing phrases as positive, negative, or neutral. In one study [21], the goal was to evaluate instructor effectiveness by identifying feedback orientation by using algorithmic supervision for sentiment analysis on feedback from students posted on a page on Facebook. A different research endeavor [22] presented a Teaching Senti-Lexicon, highlighting the advantages of teaching Senti-Lexicon over broad lexicons like SentiWordNet. It included a teaching corpus, groups, and sentiment-weighted scores and experimented using SVM, Naive Bayes and ID3 algorithms.

Sentiment analysis on student self-evaluations was used in [23] as a unique method to predict academic failure early on and support teacher intervention for improving instruction. Similarly, an algorithm based on decision trees was created in [24] to generate yearly assessment reports while considering student comments and several performance metrics. Another sentiment evaluation system presented a lexicon-based technique [25], which caused word clouds to provide visual insights into instructors’ performance by showing sentiment metrics that were highly connected with the Likert scale. In assessing student textual input, the Faculty Evaluation System (FES), divided into Sentiment Analyzer, Feature Extractor, and Feature Sentiment Evaluator, showed encouraging accuracy [26]. The usefulness of the sentiment model for classroom assessment was demonstrated by research [27] that used SMS texts to identify categories inside the words and modeled them using three different methods. The teacher’s assessment in [28] used sentiment analysis with a Naive Bayes classifier to classify comments in English and Filipino based on cumulative opinion word scores.

Extending the scope, sentiment evaluation on an educational dataset was conducted using a variety of algorithms based on machine learning and a DL technique [29], with SVM obtaining the best accuracy at 78.7%. Despite these sentiment analysis-focused efforts, several research studies have turned to aspect-based sentiment evaluation [30], which identifies and categorizes features in student input according to sentiment orientation. Further research [31] obtained noteworthy F-scores of 0.80 in extracting aspects and 0.72 in emotion detection by using dependency connections between opinion words and noun pairs for aspect extraction. Furthermore, there has yet to be much research done on DL methods in academia. Using WResNeXt-G for sentiment analysis based on aspects of student comments to assess faculty teaching ability, our work is a pioneering initiative.

### Academic excellence and data mining

The Association Rule for Mining was introduced by academics in [32] as a method for extracting valuable insights from massive datasets in the field of educational analysis of data. This method, modified for student data, is a detailed process of matching specific student attributes with organizational needs. The intricacy of this process highlights how difficult and multi-step it is. Similarly, the example study by the authors in [33] concentrated on predicting student performance using several classifiers. Four academic cohorts’ worth of data from 348 undergraduate students were examined, and the findings indicated potential for a reasonable level of accuracy. This tactic shows how committed the researchers are to looking into other ways to enhance prediction models in the educational setting. Recent work in [34] focused on predicting student accomplishment utilizing decision tree algorithms, namely BfTree, J48, and CART. Based on the results, BfTree was shown to be the most effective technique for categorization. It achieved an impressive accuracy rate of 67.07% correctly identified cases and 32.93% erroneously categorized instances. This study’s primary goal is to improve student performance by exploring the subtle variations across decision tree-based algorithms ID3, C4.5, and CART, among others. Whenever considered, these studies are all part of the continuous investigation of novel methods, such as association rule mining and decision tree algorithms, to reveal essential patterns in educational data and improve our comprehension of the prediction of student performance.

## Proposed system model

**Fig 1:**
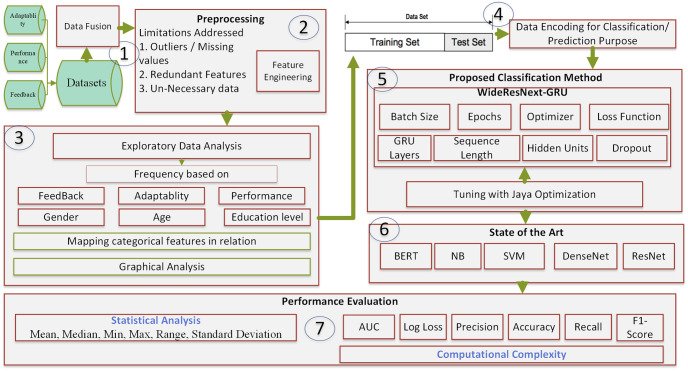
Proposed framework for student adaptability and performance.

Our study approach is carefully planned to meet specific goals, ensuring a systematic and thorough examination. We started by looking through several education datasets, choosing the one that most closely fits our study parameters, and combining many data sources to enrich our dataset. The next area of attention was preprocessing, where we carried out a thorough data purification process, carefully addressed missing values, and harmonized data formats to ensure uniformity. We implemented DL algorithms using an improved dataset, highlighting their prediction accuracy during extended training. We performed feature engineering to improve and refine variables simultaneously and optimize the dataset for the subsequent research phase. We used the DL classifier in an 80–20 train–test split to evaluate the Applicability of our Model. To show these classifiers’ Accuracy and Efficiency in achieving our study goals, algorithms were used to perform extensive training and assessment. As shown in Fig 1, this thorough approach—which included dataset exploration, collection, preprocessing, DL, and ML techniques—resulted in a robust educational evaluation.

### Datasets description

This study uses data from several Chinese schools to examine classroom performance, sentiment analysis, and flexibility. We retrieved three public datasets totaling 3,542 records using the reliable platform Kaggle [35–37]. Every dataset consists of fourteen different attributes. Among the datasets are:

- Academic performance is the focus of Dataset A, which contains 1,200 entries. Information such as participation, attendance, and grades are kept in these records.
- The second dataset is a collection of 1,142 records focusing on pupils’ adaptability degrees. Such traits include participation in extracurricular activities and the capacity to incorporate constructive criticism.
- The third dataset contains 1,200 student comments on their educational experience. Sentiment analysis is its intended use.

The data needed to be cleaned so that deep learning (DL) models could use it. This included standardizing numerical features, encoding category data, and handling missing values.

Examining the missing numbers showed that every missing data item was connected to a particular characteristic. To simplify the research, we numerically coded category categories and determined numerical values to depict performance traits and the “adaptivity level”. Numerical numbers corresponding to the “Low,” “Moderate,” and “High” categories substituted the suitable adjectives. We also used quantitative representations of positive and negative sentiments for sentiment analysis of academic performance.

### Performing preprocessing and EDA

We extensively preprocess to restore missing values, balance data, and extract features to ensure the datasets fit for deep learning methods. The following describes specific processes:

#### Handling missing values

Initially, the absent values for the academic excellence and adaptability datasets were identified and explored. Each value was examined individually to ascertain the potential impact of each lacking value on the evaluation. To preserve the dataset’s integrity and prevent the introduction of bias, we implemented average imputation for missing numerical values and median interpolation for missing qualitative variables.

- **Derivative of Mean:** In a feature *A* that had values that weren’t filled in, the missing value *A*_*j*_ was filled in with the average of the values that were filled in:
  $$\begin{array}{c} A_{j}=\bar{A}=\frac{1}{M}\sum_{j=1}^{M}A_{j} \end{array}$$ (1)
- **mode imputation**: The missing value *B*_*j*_ in a categorical feature *B* with missing values was replaced with the mode mode(*B*).
  $$\begin{array}{c} B_{j}=\text{mode}\left(B\right) \end{array}$$ (2)

#### Data balancing

Class imbalance was resolved by synthetic minority over-sampling technique (SMote). This approach generates synthetic samples in the feature space to enable a more homogeneous distribution of classes. By guaranteeing suitable representation of data from minority classes, this enhanced the performance of classification methods.

- **SMOTE:** A minority class sample *C* is used to build synthetic samples *C*_*syn*_ using the following formula:
  $$\begin{array}{c} C_{syn}=C+\zeta \cdot \left(C_{nn}-C\right) \end{array}$$ (3)
  *zeta* is a number between 0 and 1; *C*_*nn*_ is a random selected nearest neighbor.

#### Feature extraction and encoding

**Fig 2:**
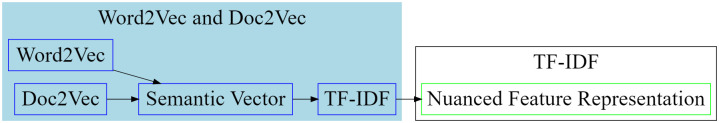
Hybrid feature extraction strategy.

Conversion of categorical variables made numeric representation in machine learning algorithms feasible. One instance is the numerical values of groups including “Low,” “Moderate,” and “High.” We employed a hybrid extracted features approach for sentiment evaluation, including TF-IDF, Doc2Vec, and Word2Vec. The hybrid feature strategy is shown diagrammatically in Fig 2.

- **Doc2Vec and Word 2Vec:** These natural language processing methods capture semantic linkages and represent words and texts vectorially.
- **TF-IDF Translation:** TF-IDF (Term Frequency-Inverse Document Frequency) lets one ascertain the relevance of the vector outputs from Word2Vec and Doc2Vec in the gathered data. This change produces a more intricate feature representation emphasizing words’ semantic links and relevance.
  $$\begin{array}{c} \text{TF-IDF}(v,g,H)=\text{TF}(v,g)\times \text{IDF}(v,H) \end{array}$$ (4)
  where
  $$\begin{array}{c} \text{TF}\left(v,g\right)=\frac{u_{v,g}}{\sum_{v^{\prime }\in g}u_{v^{\prime },g}} \end{array}$$ (5)
  and
  $$\begin{array}{c} \text{IDF}\left(v,H\right)=\text{log}\;(\frac{R}{\left|\{g\in H:v\in g\}\right|}) \end{array}$$ (6)
  *u*_*v*,*g*_ calculates the frequency of the term *v* in document *g*, *R* denotes the total count of pages and |{*g* ∈ *H* : *v* ∈ *g*}| reflects the count of documents that contain *v*.
  The hybrid model increases sentiment analysis efficiency by combining Word2Vec and Doc2Vec to preserve semantic connections and the power of TF-IDF to find significant phrases, especially for diversified datasets with complex sentiment expressions.

#### Exploratory Data Analysis (EDA)

Before creating our models, we performed a thorough exploratory data analysis (EDA) to understand better the distribution and correlations of the data’s features. We generated significant statistical measures for every property—including quantiles, averages, and standard deviations—to learn about their distribution [38].

- /textbfQuantiles: The *ϕ*-th quantile *W*_*ϕ*_ is defined for a dataset *P*-sorted in ascending order as:
  $$\begin{array}{c} W_{\phi }=P_{\left(\phi (S+1)\right)} \end{array}$$ (7)
  The desired quantile, like 0.25 for the first quartile, *S* is the total number of observations; *ϕ* is thus.
- **Mean:** The mean (*ν* of a dataset (*P* is computed using the formula.
  $$\begin{array}{c} \nu =\frac{1}{S}\sum_{j=1}^{S}P_{j} \end{array}$$ (8)
- **Standard Deviation:** There is a specific approach to calculate the standard deviation of a dataset, which is represented as *θ*.
  $$\begin{array}{c} \theta =\sqrt{\frac{1}{S-1}\sum_{j=1}^{S}{\left(P_{j}-\nu \right)}^{2}} \end{array}$$ (9)

Histograms and box plots are examples of visualization methods helpful in identifying trends, outliers, and potential correlations between variables before data processing and model creation. Our research’s accuracy and dependability were enhanced by carefully cleaning the datasets and were suited for deep learning algorithms.

### Using correlation matrix to examine relationships

**Fig 3:**
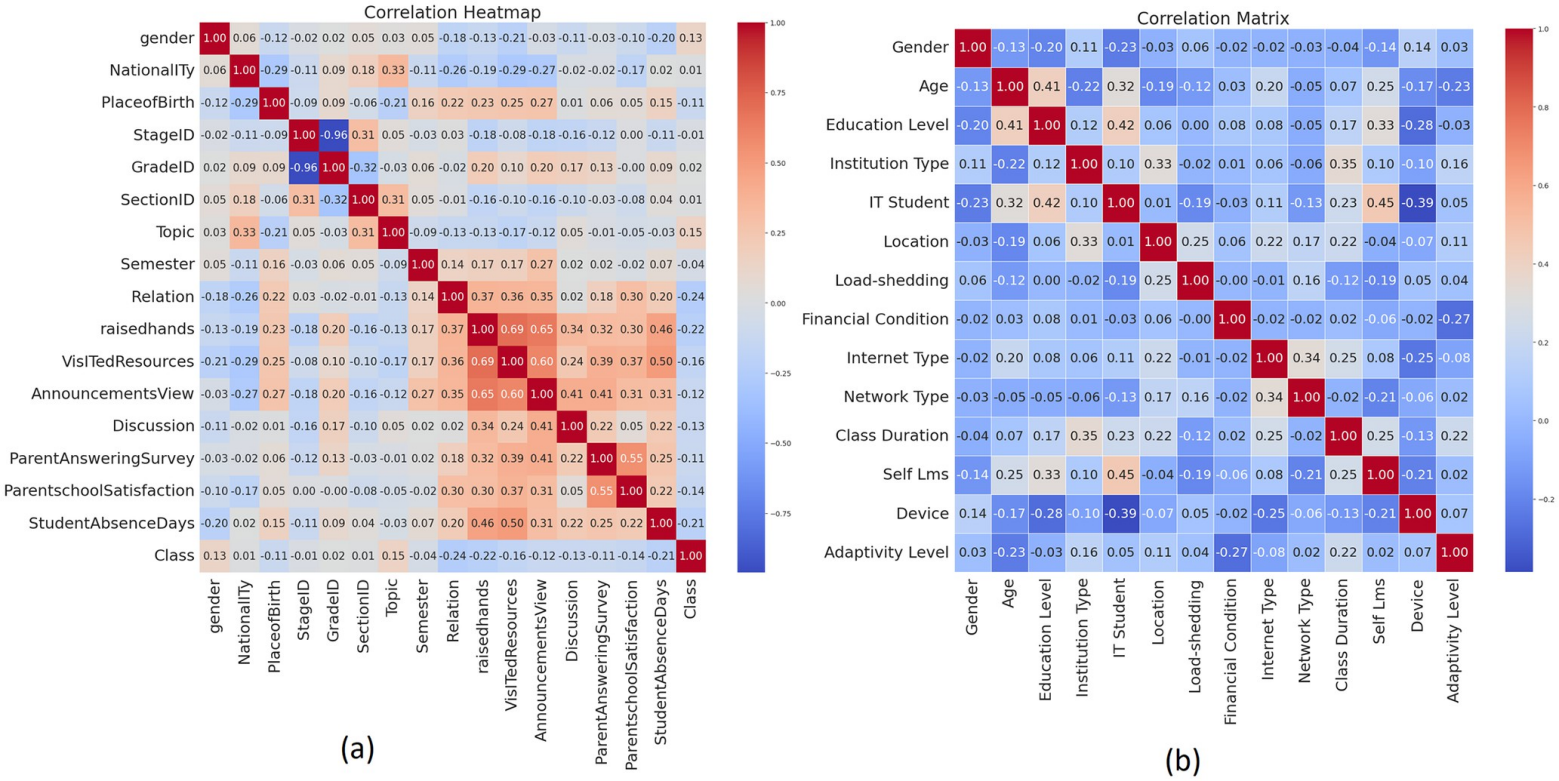
Correlation Matrix on (a) Student Performance and (b) Adaptability Dataset.

Using the appropriate color scheme and range of numbers, a matrix correlation illustrates the direct relationship between the qualities. In-depth explanations of the relationships between variables are given, surpassing simple data visualization. The correlation coefficients displayed in this matrix aid in analyzing complex relationships and patterns between variables by indicating the direction and degree of linear interactions. This analytical tool is essential for data analysis in many fields, mainly, but not limited to, financial and scientific research, because it helps with tasks like choosing features and data exploration. Fig 3 display the correlation matrix, which significantly illustrates these inter-variable interactions.

### Proposed WResNeXt-GMJ

**Fig 4:**
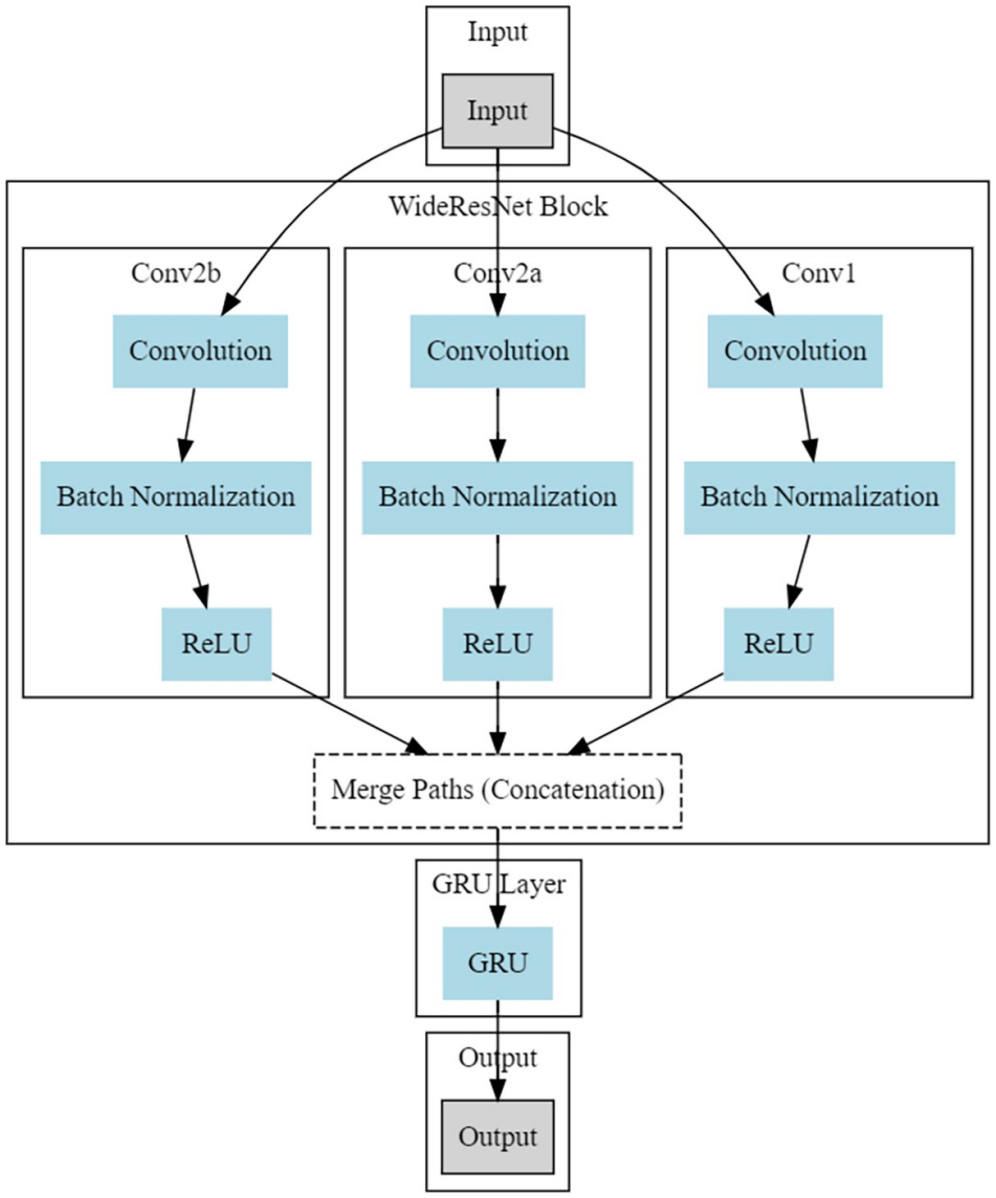
Proposed WResNeXt-G model.

To establish adaptable learning environments conducive to academic success, our DL method is grounded in the WResNeXt-G paradigm. This model showcases enhanced adaptability through the fusion of robust feature extraction inherent in the Wide ResNeXt architecture, incorporating context-based and sequential learning capabilities facilitated by GRUs. Fig 4 shows the organizational architecture of the WResNeXt-G Model and its internal structure. This amalgamation of design principles holds significant promise for crafting versatile learning environments, ultimately fostering improvements in academic achievement and flexibility.

**WideResNeXt [39]:** WideResNeXt performs feature extraction in raw student performance data and can be applied to adaptive learning environments. This is done for every route I in the WideResNeXt block; it passes through these three primary phases to help this process.

1. The Convolutional layer(*Convi*) plays a pivotal role in the feature extraction process, laying the groundwork for more in-depth explorations of student performance data (x).
2. Batch Normalization Method Data is passed through batch normalization *BNi* after the convolutional layer. Standardization and optimization of the data raise its value for possible LRPs.
3. The Rectified Linear Unit activation coefficient (*ReLU*_*i*_) enhances the data, boosting its non-linear recognized activation function and significantly improving the model’s ability to detect intricate relationships and patterns in the data.

Student Learning Adaptability and Performance (SLIP) forms the input data for the variable *x*. The architecture of the algorithm depends critically on the convolutional layer *Conv*_*i*_ as it effectively gathers data on students’ performance and adaptability in preparation of analysis. The data is then processed by the aggregate *normalization*_*i*_ and ReLU activation function(*ReLU*_*i*_) due to which. This concatenation enables the data to be suited for processing down the path I of the WideResNeXt architecture. This applies a method based on processing data, using cardinality-based multipath datasets. Because each pathway arrives at the WideResNeXt framework in isolation, SLAP characteristics can be extracted and analyzed separately.

These various methodologies will inevitably lead to different results, so the adaptation data in them might vary. Concatenation—Many outputs are still concatenated to give a logical conclusion. In this example, each path’s findings (*ReLUi*) are successfully combined using concatenation, fusing their informative data into an all-encompassing feature representation. Furthermore, this technique integrates the results of four distinct paths. Concatenated features go via a Gated Recurrent Unit (GRU) layer before reaching the critical phase of the model. This layer helps the model identify SLAP-related emergent patterns and sequential links by adding a time component to the study. The specifics of the mathematical formulas controlling the GRU processes are as follows. [40]:

$$\begin{array}{c} p=\sigma (U_{p}\cdot \left[\text{Concatenation},q_{t-1}\right]+b_{p}) \end{array}$$ (10)

$$\begin{array}{c} v=\sigma (U_{v}\cdot \left[\text{Concatenation},q_{t-1}\right]+b_{v}) \end{array}$$ (11)

$$\begin{array}{c} {\tilde{q}}_{t}=\text{tanh}\left(W\cdot \left[\text{Concatenation},p\cdot q_{t-1}\right]+b\right) \end{array}$$ (12)

$$\begin{array}{c} q_{t}=\left(1-v\right)\cdot q_{t-1}+v\cdot {\tilde{q}}_{t} \end{array}$$ (13)

These mathematical expressions describe the working principles of the GRU layer. Here, the information flow and gating procedures are managed by the unique gates ‘p’ and ‘v,’ and the variable ‘qt’ denotes the hidden state at a particular time.” Combined with these processes, the model can better adapt quickly to changing SLAP-related inputs.

### Modified Jaya Algorithm (MJA)

Optimizing hyperparameters is a crucial initial step towards improving machine learning models. These characteristics substantially impact the Model’s Accuracy, expansion, and convergence. The crucial hyperparameters meticulously defined and configured concerning SLAP utilizing our WideResNeXt-GRU model are listed in Table 2.

**Table 2:** WideResNeXt-GRU parameter values optimized by MJA.

Hyperparameter	Value achieved
Rate_leanring	0.0005
Size of Batch	128
Hidden GRU Units	256
WideResNeXt Config Block	3 blocks, depth 16, width 8
Dropout Rate	0.2
Weight Decay	0.0002
Epochs	40

**An analysis employing the Modified Jaya Algorithm [41]:** In this study, the parameters are optimized strategically by applying the Modified Jaya Algorithm. This iterative optimization strategy, motivated by collaborative population dynamics and exploring the hyperparameter space, is based on an impartial mechanism that assesses the model’s functionality on a validation set. Algorithm 1 provides a detailed description of each stage in this optimization.

**Algorithm 1** Modified Jaya Optimization Algorithm for Tuning Hyperparameters

1: **Input**:

2: Basilinec parameter settings *Q*

3: Specifying the Obj function *f*(configuration)

4: Range of every [*KY*] hyperparameter

5: *γ* is a convergence limit

6: **Output**: optimum baseline parameters

7: **procedure** Optimization_Modified Jaya

8:  Set up *P* in instances that control variables specified at arbitrary.

9:  Set up the optimal arrangement first. *x*_best_ through *Q*

10:  **While** Not matched **do**

11:   **for** every configuration *S*_*n*_ in *Q* **do**

12:    Generate at random numeric. properly with *rn* over the interval of the [0, 1]

13:    configuration Modification:

14:     *S*_*n*_ = *S*_*n*_ + *rn* ⋅ (*s*_best_ − *s*_*n*_)

15:    Verify that the parameters are in the specified span:

16:     *s*_*n*_ = min(*Y*, max(*K*, *x*_*n*_))

17:   **end for**

18:   Determine which configuration, *x*_best_, has the highest value of the objective function.

19:  **end while**

20: **end procedure**

The SLAP prediction is enhanced and produces reliable, accurate findings, and optimization is used to achieve the most precise outcomes possible. The WideResNeXt-GRU model and the Modified Jaya algorithms’ phases for managing optimizations are integrated into the suggested hybrid technique, or WResNeXt-GMJ. This integration increases the model’s efficiency and accuracy in forecasting SLAP since it is designed hybrid to fit the specific needs of every student. Algorithm 2 contains a description of the whole WResNeXt-GMJ hybrid model methodology.

**Algorithm 2** Integrated Model for SLAP

1: **procedure** PredictSLAP(Student Information)

2:  **Data Preprocessing:**

3:  Effectively deal with outliers and missing data

4:  Eliminate extraneous features

5:  Utilizing the WResNeXt-G model for feature selection

6:  **Exploratory Data Analysis (EDA):**

7:  Carry out a descriptive examination.

8:  Find commonalities across classification attributes

9:  Visualize Dataset

10:  **OptimiOptimizationss using Modified Jaya (J):**

11:  Commence with a diverse student cohort with hyperparameters

12:  Evaluate model performance leveraging the WResNeXt-G approach

13:  Select the most resilient pupils for future generations

14:  To improve, use mutation and crossover procedures.

15:  Iteratively adjust hyperparameters during the growth phase

16:  Engage in competitive selection for model survival

17:  Carry on for an initial set number of iterations

18:  **Performance Evaluation:**

19:  Divide the dataset in half to use for testing and training.

20:  Use the training data to train the WResNeXt-GMJ model.

21:  Use a variety of measures to assess the model’s efficiency.

22:  Apply statistical tests

23:  Determine measures of computational complexity

24:  **Output:** WResNeXt-GMJ for Predicting SLAP

25: **end procedure**

### Metrics for predicting learning performance and adaptability

This work predicts students’ learning potential by evaluating and forecasting their upcoming performance based on historical performance data. The investigation incorporates numerous parameters, as discussed in the dataset section. Using these parameters, student performance is predicted. To assess whether our method is functioning properly, several performance metrics are employed to verify the authenticity of our work.

#### Dynamics of accuracy and recall

Explore the complex domain of evaluating models that forecast students’ flexibility in learning, where memory and accuracy become the primary performers of assessment. Compared to the magnificence of an effectively performed assessment, these steps elegantly negotiate complexity to ensure that the model not only forecasts possible outcomes but also recognizes the validity of advantageous modifications. Accuracy expertly distinguishes between the accurate positive adaptation (TRPT) situations and the fake positives (FLPT) that are masquerading as them, giving the assessment ensemble an appearance of refinement. In the meantime, it also upfront outputs any rare fake negatives (FLNT) and switches across reality, smoothing all TRPT occurrences. Suppose we unwind the mathematical expressions in formulas 14 and 15. Then, some fine-grained details about this performance are revealed [37]. Furthermore, it added that terms of evaluation like false positives, true negatives, and true positives appropriately describe the model’s accuracy and recall over an extensive range of learning prediction phases with exceptional flexibility.

$$\begin{array}{c} \text{Precision}=\frac{\text{TRPT}}{\text{TRPT}+\text{FLPT}} \end{array}$$ (14)

$$\begin{array}{c} \text{Recall}=\frac{\text{TRPT}}{\text{TRPT}+\text{FLNT}} \end{array}$$ (15)

These trends help to evaluate the prediction power of the model and identify areas needing greater study and improvement.

#### ROC and AUC trade-off analysis

The ROC curve, which represents TRPT and the opposite of matrix dimension (TRNT) as ratios, is used to assess how well the model can distinguish various levels of learning adaptability. The section under the curve (AUC) [41] is undoubtedly a gauge for your round bias potential from the model, showing its promising potential. The FLPT rate (FLPTR) is one of the two metrics that show how many students who lack high adaptability are predicted to have it within LOAF. Instead, the TRTP Rate (TRPTR) tells us how often we find specially fitted people.

This focuses on the approach’s capacity to distinguish between multiple stages of learning adaptation while allowing a comprehensive exploitation measure. This is achieved through meticulous research, ROC curve analysis, and computing AUC. Applying one AUC value will increase its capability to defeat negative results thanks to a more adaptative strategy-based model selection. All this self-reflection is a significant part of the basis for our assessments. Our system could predict whether students would do well. The paper also includes TRPTR, FLPTR and more neutral tests.

#### Log loss evaluation

The accuracy of our model is one of the major factors affecting how well it will predict a given student’s likelihood to beat log loss when learning. Inspect the produced estimate 16 [42] to see how much, on average, the predicted probability is close (or far away) from accurate labels.

$$\begin{array}{c} \text{log}\;\text{Loss}=-\frac{1}{Z}\sum_{i=1}^{Z}(s_{n}\;\text{log}\left(m_{n}\right)+\left(1-s_{n}\right)\;\text{log}\left(1-m_{n}\right)) \end{array}$$ (16)

This formula subtracts the total number of instances (Z) from the actual classification label (*s*_*n*_), which may have values of 0 or 1, as well as the anticipated change (*m*_*n*_) that the i-th case would be determined to be exhibiting a favorable learning adaption and performance.

Log losses are computed to evaluate the accuracy of our model’s fit with actual labels and probability forecasts. In SLAP, a more consistent and dependable prediction is defined by a lesser log loss, which implies that the observable and intended labels are more in agreement.

#### Statistical examination

This paper compares the suggested hybrid approach with other approaches or baseline models using a comprehensive statistical analysis. Analysis of variance (ANOVA), student’s t-test, median deviation, average, and range are among the techniques this study uses to examine data variety and significance. One must thoroughly study computational complexity to determine the computer resources required to implement the hybrid strategy. In this work, we investigate the Scalability and usefulness of the paradigm.

Based on statistical studies that include these assessment features, the suggested hybrid approach is well-known for its accurate forecasts, resilience, and computing efficiency, which help one anticipate students’ adaptability to learning. These tests allow one to find how the model predicts classroom actions. Knowledge gained by pupils.

## Simulation results and discussion

This study was carried out on a 16GB RAM and Core i7 CPU machine under controlled experimental conditions using Python. This study uses a large dataset incorporating thoughtfully chosen from multiple SOTA [35–37] a broad spectrum of elements linked to students’ learning adaptability. After carefully reviewing the many possibilities, this dataset was selected for our work. It is fit for use in scientific environments and fulfills our objectives for study. Python’s advanced investigation tools and exceptional library support flexibility have made it the language of choice. The Core i7 CPU’s computing capability and 16 GB RAM made effective data processing, administration, modeling, and simplifying numerous analytical chores possible.

An examination was conducted in this work to ascertain the correctness of the various algorithms that are now in use within the proposed platform. The suggested strategy is the most efficient technique that guarantees precise and consistent results, according to a thorough examination. Observing this approach might improve the system’s total effectiveness and dependability.

### Data analytics

**Fig 5:**
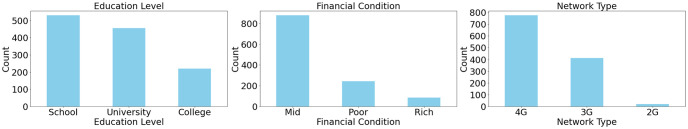
Demographic and tech insights (Adaptability).

First, This study explored the adaptability dataset. The dataset’s significant demographics and technological patterns are comprehensively summarized in Fig 5.

The gender distribution is shown in the first subplot as a horizontal bar chart with distinct bars for the categories “Boy” and “Girl.” The second subplot, which shows data for the categories “University,” “College,” and “School,” uses a horizontal bar chart to show the range of educational backgrounds. The third subplot examines internet access and displays the number of users for each category, including “Wifi” and “Mobile Data”. Fig 5 provides researchers and analysts with a practical and comprehensive way of practical visual assistance for spotting patterns and variances in gender, educational attainment, and demographic preferences for internet connections. The visualizations are easier to read when unique colors and labels are used, which makes it easier to comprehend the composition of the dataset as a whole.

**Fig 6:**
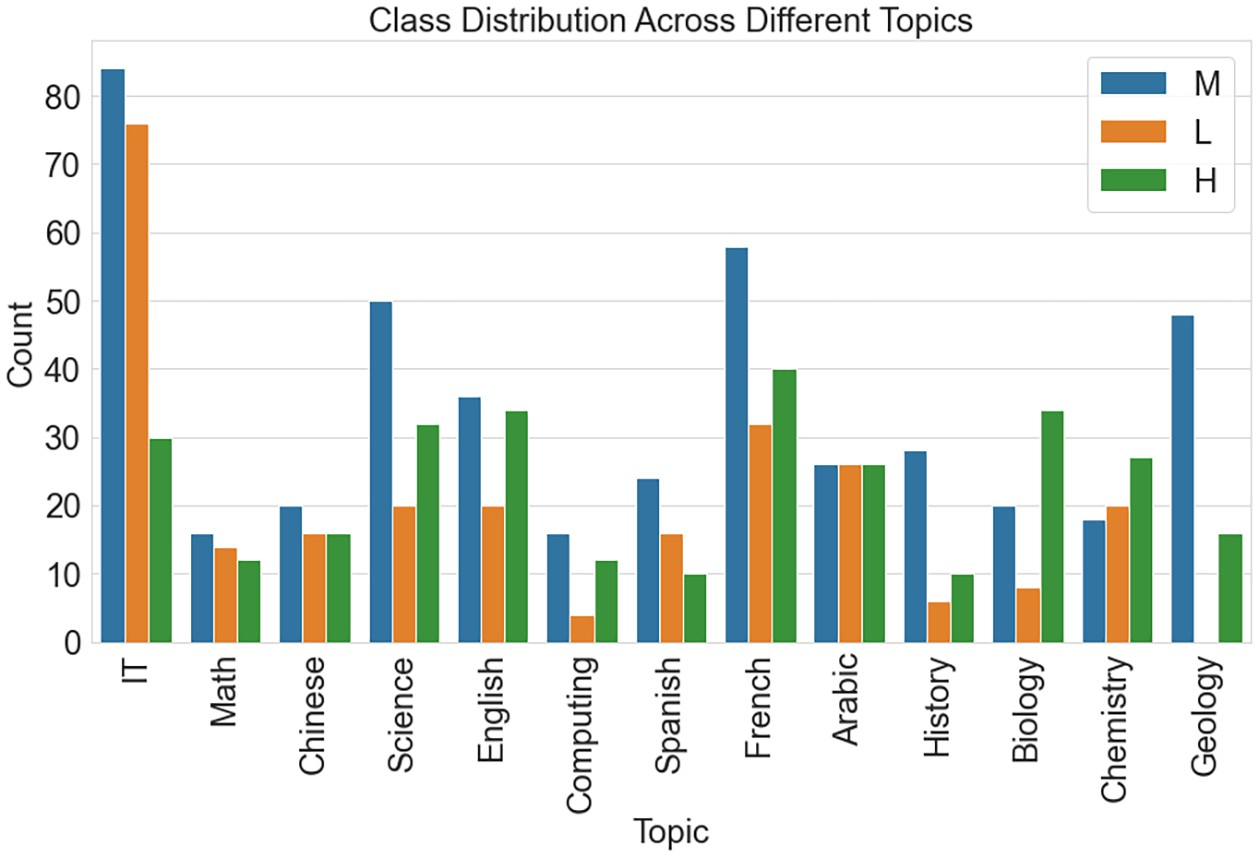
Class distribution across topics (Performance).

Fig 6 aids in identifying potential correlations or variations in class distribution across diverse topics, providing valuable insights for researchers and analysts.

**Fig 7:**
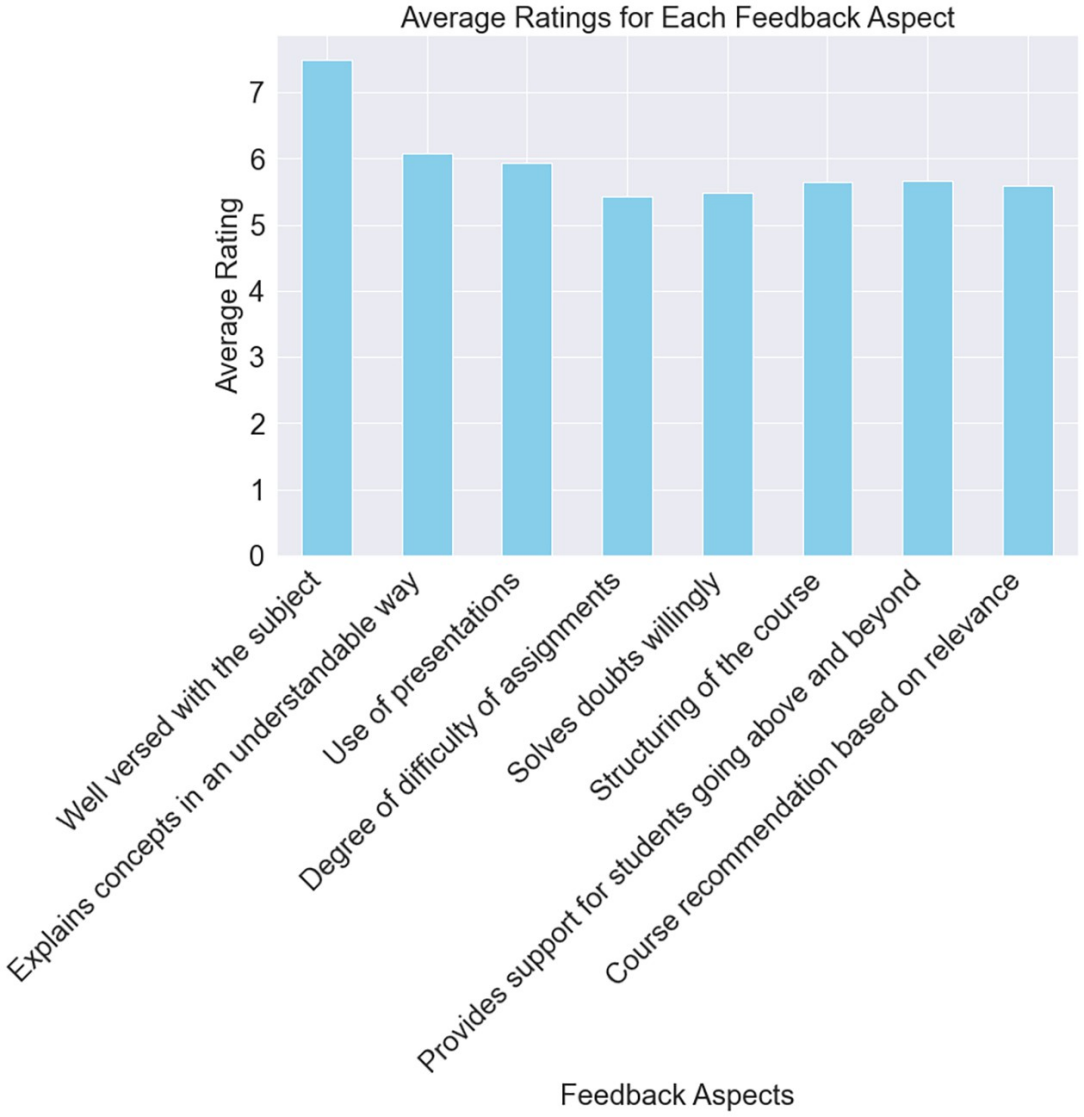
Average ratings for each feedback aspect (Students feedback).

Fig 7 shows the average scores for the various feedback sections based on the responses from the students. The feedback’s x-axis attributes include “Well-versed in the topic,” “Explains topics in an understandable method,” “Uses presentations,” and so on. The average rating for every element is shown on the y-axis.

This figure focuses on the students’ perceptions of the course’s advantages and disadvantages. Higher average ratings, for example, are probably seen favorably by the students, while lower ratings can point to areas needing development. Fig 7 helps teachers and administrators quickly discover essential topics that have a good impact on pupils and those that could need improvement. It helps with focused enhancements and course adjustments by summarizing the overall attitudes in the student feedback dataset.

**Fig 8:**
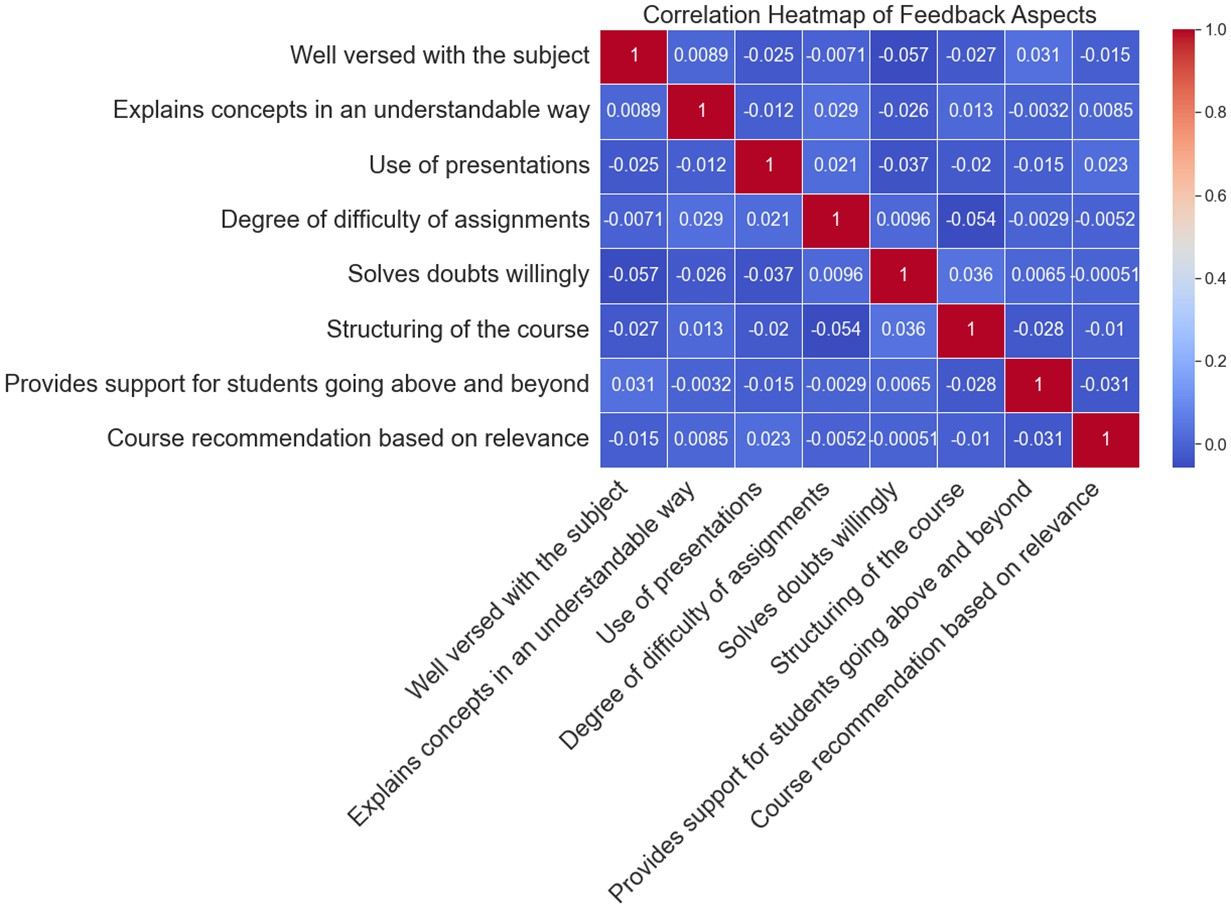
Correlation heatmap of feedback aspects (Students feedback).

In Fig 8, the correlation matrix of the different regions of student feedback is shown as a heatmap. Every square in the heatmap represents the correlation coefficient between two feedback qualities; values range from -1 to 1. Monitoring this heatmap makes it possible to identify probable correlations or connections between different areas of student input. Better ratings in one area may not always correlate with higher evaluations in another, as positive correlations show. Positive correlations, on the other hand, suggest the opposite connection. It contributes to a more thorough comprehension of the complex interactions within the feedback dataset by helping researchers and educators discover important feedback characteristics that may impact one another.

### Performance of algorithms

**Fig 9:**
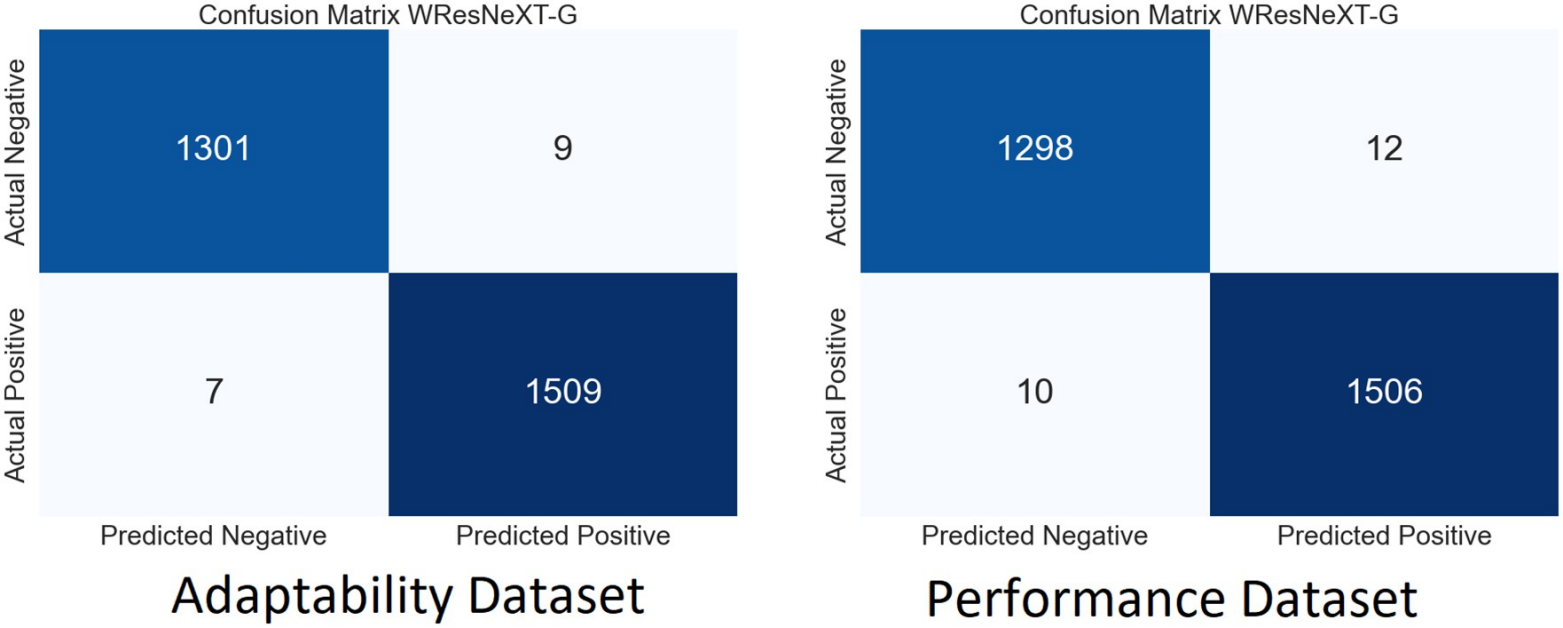
CF of WResNeXt-GMJ model.

**Fig 10:**
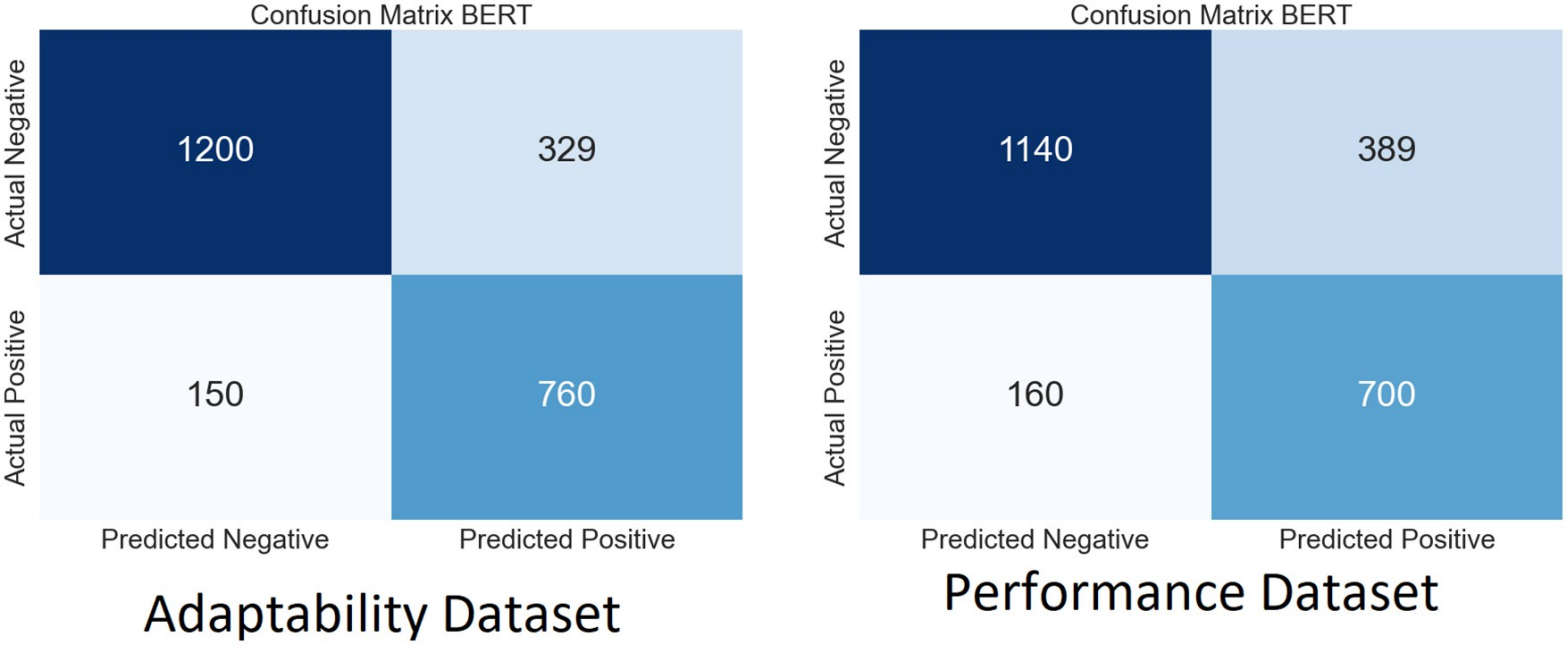
CF of BERT model.

**Fig 11:**
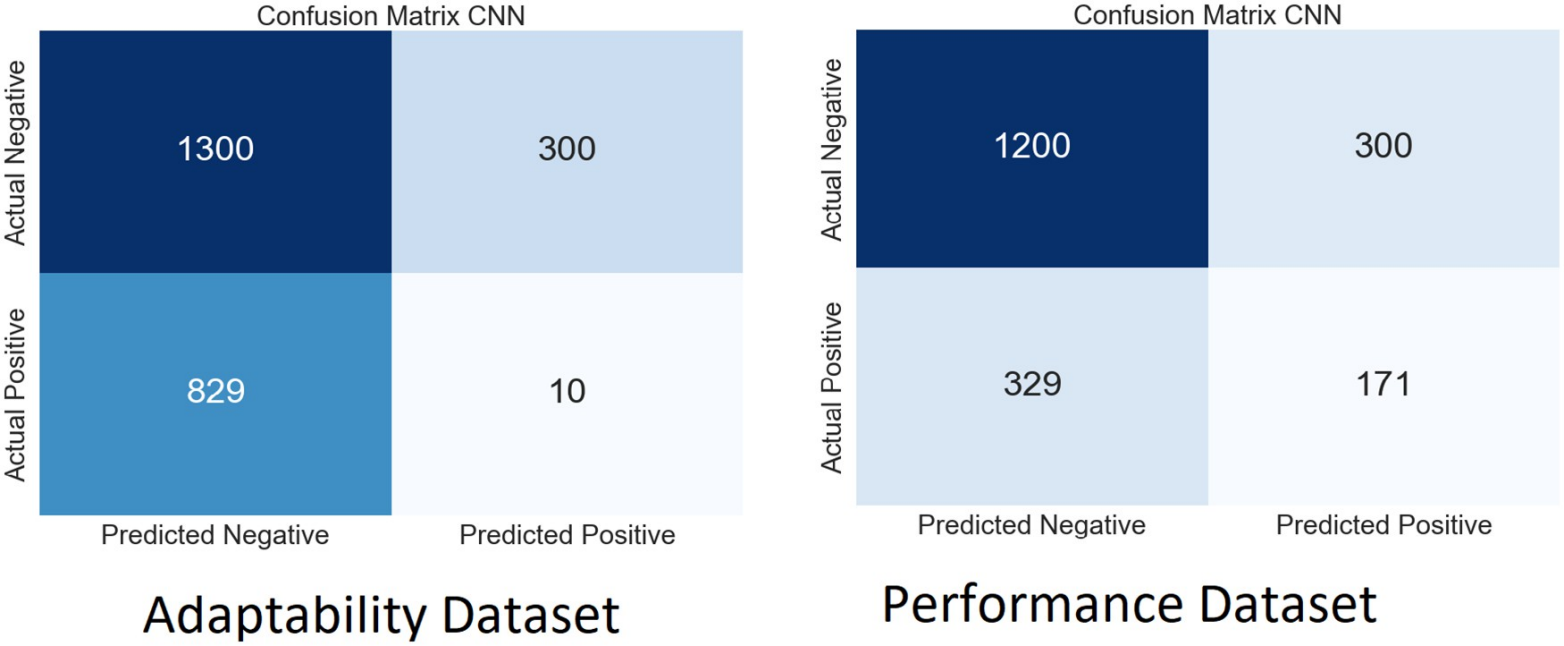
CF of CNN model.

**Fig 12:**
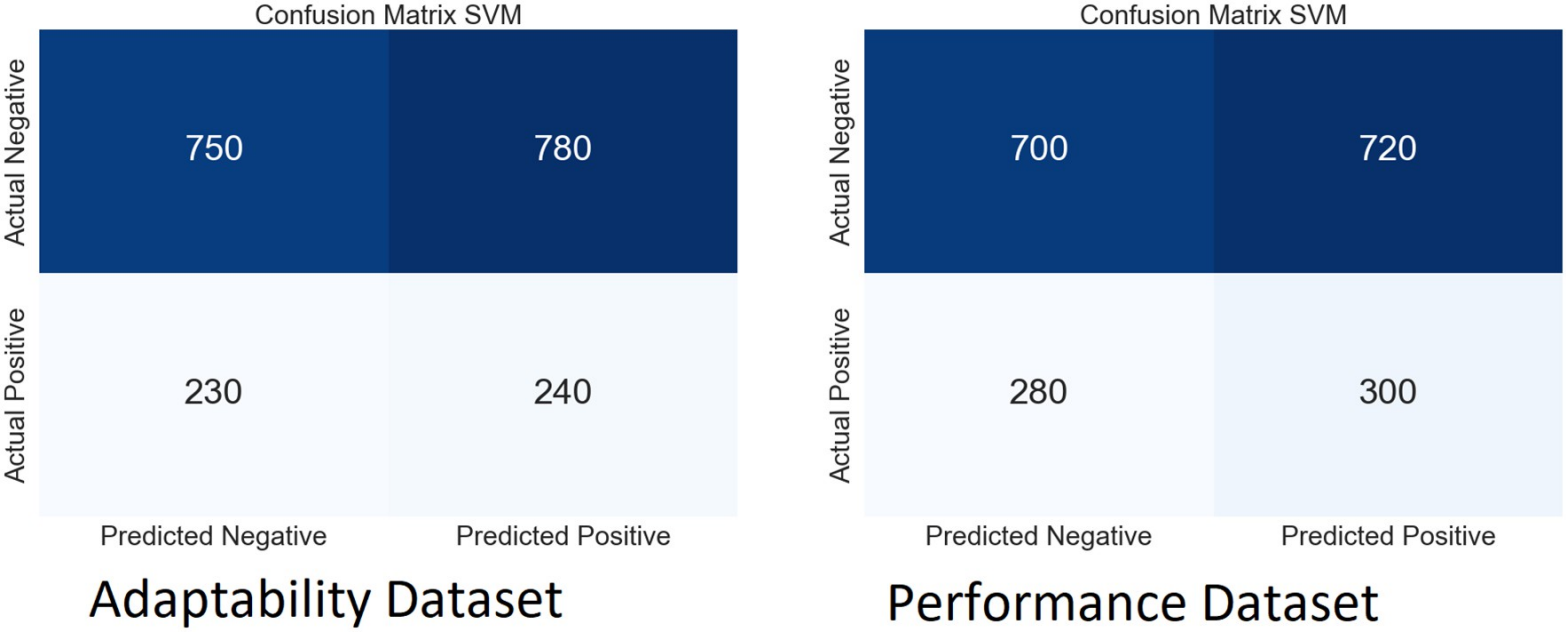
CF of SVM model.

We performed extensive simulations of our model using both SLAP datasets. Figs 9–12 show the ambiguity matrices used in the simulation. Results show that, in terms of classification methods, the WResNeXt-GMJ Model beats both BERT, CNN, and SVM on both datasets. Regarding anticipating student learning adaptability, the model shows good performance, given its improved Efficacy in precisely spotting events.

Each subfigure inside the main figure shows the results for the same method with different datasets. Fig 9 show the confusion matrix on the performance and adaptability datasets of the proposed method, WResNeXt-GMJ. Furthermore, Fig 10 show the values of BERT model confusion matrix on both datasets. Fig 11 present the confusion matrices for the CNN method. Moreover, Fig 12 display the confusion matrices for the SVM method. Among all, WResNeXt-GMJ demonstrates better true positive values and superior performance.

Table 3 provides a more in-depth analysis of how the various models performed on the Adaptability Dataset. Several models are ranked and assessed using different metrics in the table’s leftmost column. Among the metrics are AUC (area under the curve), F1-Score, log loss, Accuracy, and precision. Their degree of adaptability further divides them into three groups: low, moderate, and high. Furthermore, Moderate (M) and Intermediate (I) are considered interchangeable and identical. High (H) and Advanced (A) are also interchangeable terms.

**Table 3:** Performance evaluation on adaptability dataset.

Model	Category	Log Loss	Accuracy	AUC	Precision	F1-Score	Recall
**WResNeXt-GMJ**	Low (L)	0.06	0.96	0.99	0.98	0.95	0.97
	Intermediate (I)	0.07	0.92	0.97	0.93	0.95	0.95
	Advanced (A)	0.05	0.94	0.95	0.94	0.96	0.93
ResNet [22]	Low (L)	0.53	0.88	0.86	0.94	0.90	0.90
	Intermediate (I)	0.48	0.89	0.73	0.87	0.91	0.91
	Advanced (A)	0.62	0.76	0.64	0.86	0.80	0.75
BERT [20]	Low (L)	0.73	0.73	0.79	0.66	0.75	0.86
	Intermediate (I)	0.85	0.62	0.62	0.83	0.66	0.55
	Advanced (A)	1.00	0.59	0.50	0.53	0.61	0.71
CNN [18]	Low (L)	0.59	0.77	0.70	0.75	0.79	0.84
	Intermediate (I)	0.66	0.73	0.64	0.77	0.76	0.75
	Advanced (A)	0.77	0.57	0.52	0.72	0.59	0.49
SVM [18]	Low (L)	0.42	0.82	0.76	0.86	0.84	0.83
	Intermediate (I)	0.46	0.80	0.73	0.80	0.83	0.87
	Advanced (A)	0.72	0.65	0.58	0.80	0.68	0.60
NB [28]	Low (L)	0.39	0.90	0.87	0.94	0.92	0.90
	Intermediate (I)	0.54	0.88	0.79	0.86	0.90	0.95
	Advanced (A)	0.64	0.70	0.67	0.87	0.73	0.62
InceptionV4 [43]	Low (L)	0.066	0.864	0.882	0.873	0.855	0.864
	Intermediate (I)	0.088	0.828	0.846	0.819	0.837	0.846
	Advanced (A)	0.055	0.846	0.846	0.837	0.846	0.819
DenseNet-169 [44]	Low (L)	0.099	0.819	0.828	0.819	0.801	0.828
	Intermediate (I)	0.121	0.792	0.783	0.774	0.792	0.801
	Advanced (A)	0.077	0.846	0.846	0.846	0.837	0.819

The WResNeXt-GMJ model is quite effective at any degree of adaptation. In the Low adaptability category, WResNeXt-GMJ achieves a Log Loss of 0.06 with a 0.96 accuracy, an AUC of 0.99, a precision of 0.98, an F1-Score of 0.95, and a recall of 0.97. We see the same dominance patterns at both the Moderate and High adaption levels, proving that the model can consistently and reliably make predictions. Nevertheless, various degrees of adaptation are explored using models such as ResNet, BERT, CNN, SVM, inceptionV4, denseNet, and NB. Several measures demonstrate that the WResNeXt-GMJ model outperforms its rivals in predicting students’ learning adaptability. This is very remarkable. The supplied table helps compare and examine the Efficacy of various models designed for adaption prediction on the dataset.

The performance evaluation measures for the different models on the Student Performance dataset are shown in Table 4. Every measure in every area indicates that the WResNeXt-GMJ model is top-notch. As a result of making accurate probabilistic predictions, it produces low Log Loss values. Accuracy, AUC, Precision, F1-Score, and Recall are all well achieved by the model, indicating that it has overall solid skills in classification and prediction. Surprisingly, the WResNeXt-GMJ model surpasses all other models in every metric of Accuracy and AUC. With excellent Accuracy and AUC, BERT performs well in the Low class. The model struggles in the Moderate and High categories, resulting in lower Accuracy and AUC scores. The fact that Precision, F1-Score, and Recall have all gone down indicates how difficult it is to make accurate predictions. The CNN model gets acceptable results across the board regarding AUC, Accuracy, and other measures.

**Table 4:** Performance evaluation on student performance dataset.

Model	Category	Log Loss	Accuracy	AUC	Precision	F1-Score	Recall
**WResNeXt-GMJ**	Low (L)	0.05	0.97	0.99	0.98	0.95	0.97
	Intermediate (I)	0.06	0.93	0.97	0.94	0.95	0.95
	Advanced (A)	0.04	0.95	0.96	0.95	0.96	0.93
ResNet [22]	Low (L)	0.056	0.876	0.846	0.912	0.882	0.882
	Intermediate (I)	0.051	0.783	0.639	0.765	0.801	0.801
	Advanced (A)	0.066	0.666	0.558	0.756	0.702	0.657
BERT [20]	Low (L)	0.781	0.639	0.847	0.576	0.657	0.756
	Intermediate (I)	0.913	0.543	0.432	0.459	0.531	0.621
	Advanced (A)	1.078	0.513	0.432	0.459	0.531	0.621
CNN [18]	Low (L)	0.627	0.675	0.612	0.657	0.687	0.762
	Intermediate (I)	0.704	0.639	0.558	0.675	0.666	0.657
	Advanced (A)	0.825	0.495	0.450	0.630	0.513	0.423
SVM [18]	Low (L)	0.440	0.720	0.666	0.756	0.738	0.729
	Intermediate (I)	0.484	0.702	0.639	0.702	0.729	0.765
	Advanced (A)	0.770	0.567	0.504	0.702	0.594	0.522
NB [28]	Low (L)	0.407	0.792	0.765	0.828	0.819	0.792
	Intermediate (I)	0.572	0.774	0.693	0.756	0.792	0.837
	Advanced (A)	0.682	0.612	0.585	0.765	0.630	0.531
InceptionV4 [43]	Low (L)	0.066	0.864	0.882	0.873	0.855	0.864
	Intermediate (I)	0.088	0.828	0.846	0.819	0.837	0.846
	Advanced (A)	0.055	0.846	0.846	0.837	0.846	0.819
DenseNet-169 [44]	Low (L)	0.099	0.819	0.828	0.819	0.801	0.828
	Intermediate (I)	0.121	0.792	0.783	0.774	0.792	0.801
	Advanced (A)	0.077	0.846	0.846	0.846	0.837	0.819

**Fig 13:**
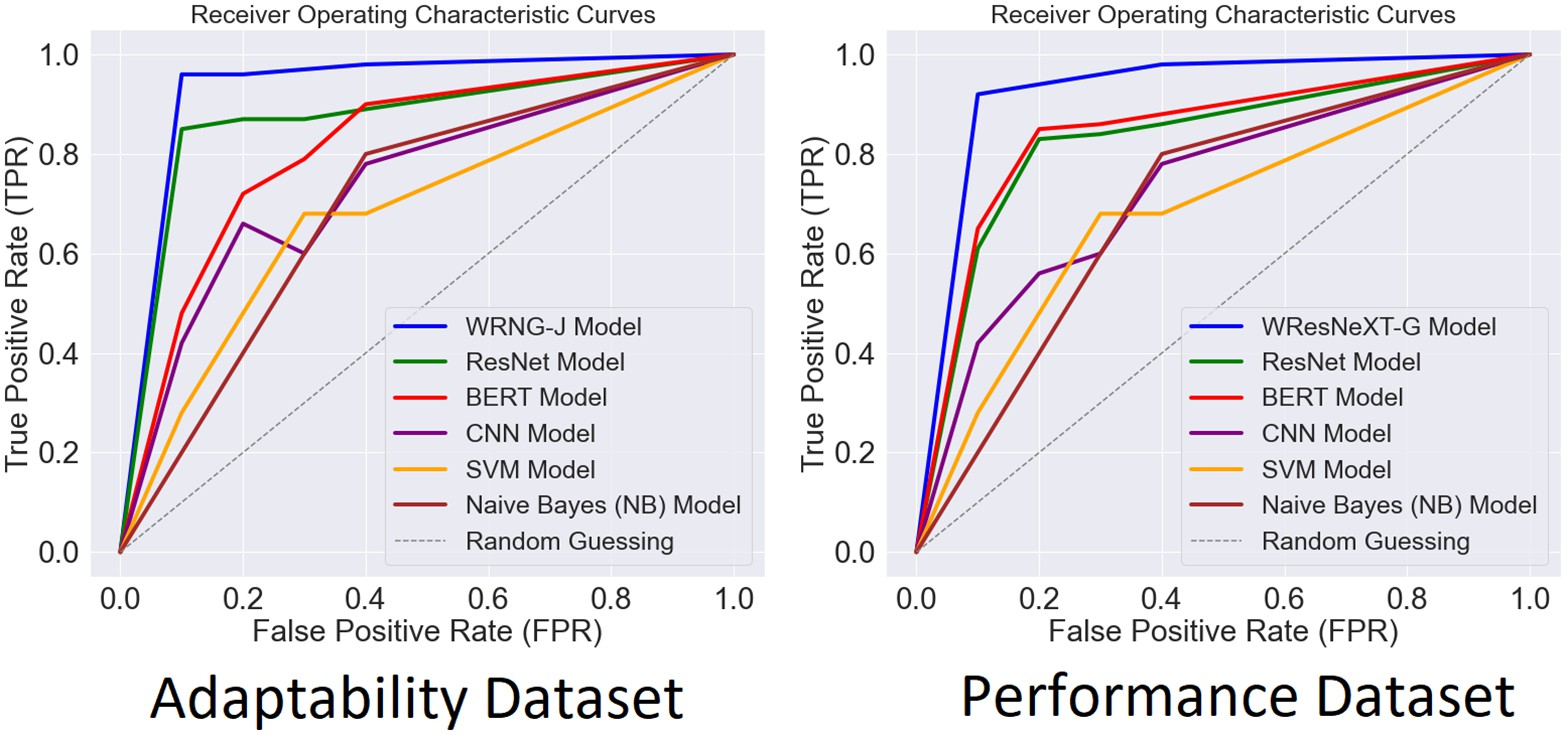
CF of SVM model.

The model’s performance over the two datasets is shown in Fig 13, which also shows the receiver characteristic (ROC) curve. With improved performance across various TPR and False Positive Rates, WResNeXt-GMJ stands out as an exceptionally successful technique.

Our unique WResNeXt-GMJ technique for predicting academic performance is highlighted in Table 5, which delves into the specifics of computational efficiency in different models. The table summarizes critical details such as the models’ average and median execution durations, ranges, and standard deviations, giving a comprehensive overview of their temporal dynamics. Each model’s average mean execution time reveals how long it takes to digest data and predict. It is hard to believe how efficient the WResNeXt-GMJ model is since its average execution time is under 37 seconds. The 88-second length is much longer than ResNet’s somewhat shorter one. In addition to being more successful than the mean, the WResNeXt-GMJ approach has a median execution time of 36 seconds, which is more resilient to extreme values and finishes tasks in 36 seconds, as opposed to 89 seconds for the CNN model. In an ideal scenario, the WResNeXt-GMJ model completes tasks in around 41 seconds, which is impressive. This model produces better results than the naïve Bayesian (NB) and support vector machine (SVM) models, which finished their tasks in 96 seconds.

**Table 5:** Execution time comparison.

Model	Min (s)	Std. Dev. (s)	Mean (s)	Max (s)	Range (s)	Median (s)
BERT [20]	74	4.21	85	96	20	86
CNN [18]	73	4.36	84	95	20	84
SVM [18]	77	3.15	85	95	16	86
ResNet [22]	72	4.39	85	95	21	88
NB [28]	74	3.95	84	96	20	83
XGB [20]	75	3.09	83	91	14	83
InceptionV4 [43]	76	2.77	82	90	14	81
DenseNet-169 [44]	75	2.51	80	87	12	79
**WResNeXt-GMJ**	34	1.11	37	41	5	36

Through a comprehensive statistical analysis, Table 6 highlights the main trends and distinctions in the effectiveness of the proposed and current strategies. In addition to offering a thorough analysis of the data and significance levels for several statistical tests, the table also allows a comprehensive assessment of the benefits and drawbacks of the various approaches.

**Table 6:** Evaluation of the WResNeXt-GMJ and current methods using statistical.

Methods		BERT		SVM		WResNeXt-GMJ		ResNet	
Tests		F-stat	P-Val	F-stat	P-Val	F-stat	P-Val	F-stat	P-Val
Correlation Tests	Kendall’s	0.554	-0.01	0.624	-0.01	0.791	-0.01	0.624	-0.01
	Pearson’s	0.624	-0.01	0.624	-0.01	0.889	-0.01	0.624	-0.01
	Chi-Squared	105.211	0.005	101	-0.01	109.43	0.043	101	-0.01
	Spearman’s	0.599	-0.01	0.624	-0.01	0.857	-0.01	0.624	-0.01
Non-Parametric Statistical Hypothesis Tests	Wilcoxon	15313.99	0.157	-0.01	-0.01	-0.01	-0.01	-0.01	-0.01
	Kruskal	6.707	0.009	24.991	-0.01	30.948	-0.01	24.991	-0.01
	Mann-Whitney	26519.99	-0.01	41231.99	0.801	26519.99	-0.01	29525	0.005
Parametric Statistical Hypothesis Tests	Student’s	-0.741	0.455	5.11	-0.01	5.721	-0.01	5.11	-0.01
	Paired Student’s	-1.078	0.286	8.421	-0.01	9.005	-0.01	8.421	-0.01
	ANOVA	0.534	0.455	26.206	-0.01	32.837	-0.01	26.206	-0.01
Non-Parametric Statistical Hypothesis Tests	Wilcoxon	-0.01	-0.01	0	-0.01	195.99	0	0	-0.01
	Kruskal	24.991	-0.01	25	-0.01	6.697	0.01	24.1	-0.01
	Mann-Whitney	-0.01	-0.01	0	-0.01	153.99	0.157	0	-0.01

A “0” p-value indicates the absence of a statistically significant impact or difference. If the p-value is more than zero but still extremely tiny, preferably less than 0.04, and the result reveals a difference or impact, it is considered statistically significant. Negative p-values are generally not regarded as necessary because of the prevalence of positive p-values. Data with negative p-values should be handled carefully while being analyzed.

In light of the above results, this research focuses on comprehending the complex elements that impact student performance, such as family dynamics, student learning strategies, and instructional approaches. Using sophisticated visualization tools and meticulous studies, we have successfully clarified intricate linkages within the data. The results of our research emphasize the substantial influence of these elements on student achievements, offering vital knowledge for improving instructional methods and promoting student flexibility. Combining data visualization and rigorous experimentation allows for a thorough study, providing detailed interpretations and practical suggestions for educators and policymakers.

#### WResNeXt-GMJ model’s incorporation into classroom practice

Our study’s practical implications for education can be realized through the strategic use of cloud computing to automate the comprehensive historical records-based, advanced analysis of student data. First Stage combining different datasets, from student performancestry to signals of adaptability; Robust pretreatment: 12 techniques for standardization, categorical encoding and handling missing values The preparation process is instrumental in enabling more advanced analytics. It makes sure that the data is consistent and does not lose its integrity. At this point, it needs to be able to evaluate and allocate the right amount of computational resources most ideally ensure utilization using scalable cloud infrastructure. Understand that by collaborating with the IT departments, our WResNeXt-GMJ model is already LMS compatible.

This technology integration allow the companies do whatever they dreams to make execute their vision with cloud computing. It is a technology that enables educators to analyze student data in real time and thereby create personalized learning interventions/ adapt lessons as needed. The high level of automation built into cloud-based deployments are naturally beneficial for handling this considerable heterogeneity in student demand and institution profile.

The steady performance of the model helps us work on boosting its predictive ability and promoting its applicability in educational settings. Educational institutions are using cloud technology to make data analysis easier and more specific in relation to student outcomes and instruction delivery, supporting a move towards a Data-Driven strategy.

## Conclusion and future directions

Emphasizing the development of academic flexibility and the generation of performance projections by feedback analysis, this work has tremendously benefited the fields of EDM and DL. This paper demonstrates how one may tailor deep learning approaches to overcome specific educational challenges by using the WResNeXt-GMJ model and other advanced algorithms. The careful study of the many applications of deep learning highlights the pragmatic adaptability of machine learning in the classroom. In this study, the WResNeXt-GMJ model is renowned for its great flexibility and accuracy in assessing student performance and mood. This success shows how precisely deep learning can generate using intricate computational approaches instructive notions. First one must understand the limitations this research imposed. Important knowledge gaps like the interpretability of deep learning models, educational environment variation, and data accessibility need further study. By means of ongoing investigation of fresh data sources and technique refinement, one may overcome these limitations and improve the resilience and flexibility of prediction models. The study also emphasizes the importance of optimizing techniques for improved projections. The proposed architecture gives careful data preparation utilizing datasets from Chinese institutions top priority in ensuring the authenticity and accuracy of forecasts. One methodological approach of this study is to combine Word2Vec, Doc2Vec, and TF-IDF into a hybrid feature extracting system. This approach broadens and deepens sentiment evaluation and helps one better grasp student feedback dynamics. This paper clarifies our knowledge of artificial intelligence’s influence in the classroom by offering doable suggestions for increasing students’ adaptability and performance. With innovative deep learning algorithms and creative approaches, this work seeks to enhance future personalized learning environments and integration of educational technologies.
