# Correction: Innovative models for enhanced student adaptability and performance in educational environments

**DOI:** 10.1371/journal.pone.0316292

**Published:** 2024-12-19

**Authors:** Lanbo Liu, Lihong Wan

The Funding statement for this paper is incorrect. The correct Funding statement is as follows: This paper is funded by the Scientific Research Project of Tianjin Municipal Education Commission ’Transformation and System Construction of Labor Education in Colleges and Universities in the Era of Digital Intelligence’ (Project No.: 2023SK055).
